# Supplementary material for: Genetic Diversity in the Diminazene Resistance-Associated P2 Adenosine Transporter-1 (AT-1) Gene of Trypanosoma evansi
Source: Animals (Basel). 2025 Mar 6;15(5):756. doi: 10.3390/ani15050756 (PMC11898887; doi:10.3390/ani15050756)
Supplement: Supplementary file 1 [file animals-15-00756-s001.zip › animals-3485066-supplementary.pdf]

Table S1. Primer set for *T. evansi* AT-1 locus amplification. TbAT _For/TbAT _Rev primer sequences are underlined, N's are bolded.

Sequences (5′-3′)*.	Primer Name..
TCGTCGGCAGCGTCAGATGTGTATAAGAGACAGCGCCGCACTCATCGCCCCG*T*T.	TeAT_For..
TCGTCGGCAGCGTCAGATGTGTATAAGAGACAG**N**CGCCGCACTCATCGCCCCG*T*T.	TeAT_For-1N ..
TCGTCGGCAGCGTCAGATGTGTATAAGAGACAG**NN**CGCCGCACTCATCGCCCCG*T*T.	TeAT _For-2N ..
TCGTCGGCAGCGTCAGATGTGTATAAGAGACAG**NNN**CGCCGCACTCATCGCCCCG*T*T.	TeAT _For-3N ..
GTCTCGTGGGCTCGGAGATGTGTATAAGAGACAGGCGAAGTACACGGCAGGG*T*A.	TeAT _Rev..
GTCTCGTGGGCTCGGAGATGTGTATAAGAGACAG**N**GCGAAGTACACGGCAGGG*T*A.	TeAT _Rev-1N ..
GTCTCGTGGGCTCGGAGATGTGTATAAGAGACAG**NN**GCGAAGTACACGGCAGGG*T*A.	TeAT _Rev-2N ..
GTCTCGTGGGCTCGGAGATGTGTATAAGAGACAG**NNN**GCGAAGTACACGGCAGGG*T*A.	TeAT _Rev-3N ..

* TbAT _For and TbAT _Rev primer sequences underlined, N's in bolded.

Table S2. Deep amplicon sequencing data of haemoprotozoan parasites from field samples. A total of 26 *Trypanosoma*-positive samples [cattle (n = 14), buffalo (n = 2), camel (n = 7), sheep (n = 2), goat (n = 1)] were collected from veterinary clinics throughout the Punjab province of Pakistan.

Field Isolates.	Host.	Endemic region.	*T.evansi* reads..
Pop203.	Camel.	Rahim Yar Khan.	1237..
Pop207.	Camel.	Rahim Yar Khan.	1436..
Pop266.	Camel.	Multan.	42556..
Pop277.	Buffalo.	Layyah.	54269..
Pop278.	Buffalo.	Layyah.	73761..
Pop279.	Cattle.	Layyah.	83134..
Pop287.	Goat.	Rahim Yar Khan.	36683..
Pop100.	Cattle.	Bahawalpur.	1347..
Pop111.	Cattle.	Bahawalpur.	1158..
Pop112.	Cattle.	Lodhraan.	1471..
Pop202.	Camel.	Rahim Yar Khan.	1457..
Pop208.	Camel.	Rahim Yar Khan.	1291..
Pop210.	Camel.	Rahim Yar Khan.	1546..
Pop268.	Cattle.	Layyah.	80011..
Pop283.	Sheep.	Rahim Yar Khan.	51373..
Pop284.	Sheep.	Rahim Yar Khan.	66216..
Pop253.	Cattle.	Muzafargar.	1964..
Pop199.	Camel.	Lodhraan.	1651..
Pop234.	Cattle.	Bahawalpur.	1686..
Pop217.	Cattle.	Dera Ghazi Khan.	1561..
Pop220.	Cattle.	Dera Ghazi Khan.	1912..
Pop237.	Cattle.	Bahawalpur.	4700..
Pop230.	Cattle.	Dera Ghazi Khan.	1259..
Pop238.	Cattle.	Dera Ghazi Khan.	5693..

Pop25.	Cattle.	Muzafargar.	1158..
Pop114.	Cattle.	Rahim Yar Khan.	1237..

Table S3. Deep amplicon sequencing data of adenosine transporter-1 (AT-1) locus in 26 *T. evansi* isolates.

..	.		Resistance type reads (individual allele)..									
**Field isolates.**	**Total no of Illumina MiSeq reads.**	**Susceptible type reads.**	**GAA (178E).**	**TCA (178S).**	**GGT (239Y).**	**GCT (239A).**	**GAG (239E).**	**AGC (286S).**	**CAC (286H).**	**ATC (286I).**	**GAC (286D).**	**ACC (286T)..**
Pop203.	2675.	1954..	.		189..	.		245.	287..	.	.	.
Pop207.	2261.	1676.	.	.	.	.	.	.	329.	256.	.	..
Pop266.	2716.	1851.	.	.	.	245.	.	.	.	276.	165.	179..
Pop277.	2560.	1771.	158.	123.	.	143.	365.	.	.	.	.	..
Pop278.	1426.	1103..	.		145..	.		178..	.	.	.	.
Pop279.	1409.	1230.	.	.	.	.	.	.	.	.	179.	..
Pop287.	1954.	1440.	.	.	199.	.	.	.	.	.	166.	149..
Pop100.	1262.	1262..	.	.	.	.	.	.	.	.	.	.
Pop111.	1397.	1397..	.	.	.	.	.	.	.	.	.	.
Pop112.	1346.	1346..	.	.	.	.	.	.	.	.	.	.
Pop202.	2361.	2361..	.	.	.	.	.	.	.	.	.	.
Pop208.	2733.	2733..	.	.	.	.	.	.	.	.	.	.
Pop210.	1960.	1960..	.	.	.	.	.	.	.	.	.	.
Pop268.	1509.	1509..	.	.	.	.	.	.	.	.	.	.
Pop283.	1708.	1708..	.	.	.	.	.	.	.	.	.	.
Pop284.	1444.	1444..	.	.	.	.	.	.	.	.	.	.
Pop253.	1614.	1614..	.	.	.	.	.	.	.	.	.	.
Pop199.	2261.	2261..	.	.	.	.	.	.	.	.	.	.
Pop234.	2221.	2221..	.	.	.	.	.	.	.	.	.	.
Pop217.	1289.	1289..	.	.	.	.	.	.	.	.	.	.
Pop220.	2282.	2282..	.	.	.	.	.	.	.	.	.	.
Pop237.	1247.	1247..	.	.	.	.	.	.	.	.	.	.
Pop230.	2267.	2267..	.	.	.	.	.	.	.	.	.	.
Pop238.	2843.	2843..	.	.	.	.	.	.	.	.	.	.
Pop25.	3087.	3087..	.	.	.	.	.	.	.	.	.	.
Pop114.	3276.	3276..	.	.	.	.	.	.	.	.	.	.
